# Normative Data for the D-KEFS Color-Word Interference and Trail Making Tests Adapted in Greek Adult Population 20–49 Years Old

**DOI:** 10.3390/neurosci5040029

**Published:** 2024-10-04

**Authors:** Marianna Tsatali, Fotini Surdu, Andromachi Konstantinou, Despina Moraitou

**Affiliations:** 1 Laboratory of Psychology, Department of Cognition, Brain and Behavior, School of Psychology, Aristotle University of Thessaloniki (AUTh), 54124 Thessaloniki, Greece; demorait@psy.auth.gr; 2 Department of Psychology, School of Humanities and Social Sciences, University of Western Macedonia, 50100 Kozani, Greece; fotini.surdu@outlook.com (F.S.); andromachikons@gmail.com (A.K.); 3 Laboratory of Neurodegenerative Diseases, Center for Interdisciplinary Research and Innovation, Aristotle University of Thessaloniki (CIRI-AUTh), 54124 Thessaloniki, Greece

**Keywords:** D-KEFS, color-word interference, executive functions, normative data, trail making test

## Abstract

Background: This study was designed to adapt the Delis–Kaplan Executive System (D-KEFS) version of the Color-Word Interference (CWIT) and Trail Making Tests (TMTs) for the Greek adult population from 20 to 49 years old, since it is of research as well as clinical importance to detect executive functions’ impairment in young adults with neurological or/and psychiatric conditions. Aims: Norms for the Greek adult population have been calculated to be available for neuropsychologists and health professionals who work in relevant settings. Methods: The study sample consisted of 101 healthy adults (41% male and 60% female) aged 20 to 49 years (M = 32.16, SD = 11.57) with education from 12 to 19 years of schooling (M = 14.51, SD = 0.89). A Pearson correlation test as well as a chi square test were conducted to examine potential associations between gender, age, education, and participants’ performance. Afterwards, we calculated normative data using raw scores and transformed them into percentile scores. Finally, Greek norms were compared to the original raw scores, which were transformed into scaled scores by Delis et al. (2001). Results: The findings showed that age was the only variable which affected CWIT, whereas level of education as well as age were predictive factors for most TMT conditions, except for the visual scanning test (Condition 1). Gender did not affect both tests. Finally, D-KEFS norms for CWIT and TMT are available for the Greek adult population to help clinicians detect executive functions’ deficits and therefore adjust tailored therapeutic strategies. Additionally, it is of great importance to use these tests for research purposes. Conclusion: Given that executive functions are assumed as high-level skills, which are highly related to everyday functionality, adapted tests contribute not only to assess the progression of any existing neurological as well as psychiatric disorders, but they can also be used to evaluate patients’ ability to live independently, as well as their access to work.

## 1. Introduction

### 1.1. Basic Characteristics of the Delis–Kaplan Executive Function System (D-KEFS)

Executive functions constitute an active functional system, with various meaningful domains: working memory, planning, fluency, inhibition, and set shifting [1]. They are also defined as a set of control processes that enable individuals to manage and direct their attention, thoughts, and actions to achieve adaptive goals [2]. According to Diamond (2013) [3], executive functions are also divided in three main categories: working memory, through updating, inhibiting and shifting, the individual keeps the information active from attentional distraction [4]; inhibition, where the individual deliberately inhibits dominant or automatic impulses when necessary [4]; and shifting, in which the individual is able to alternate and shift between tasks or mental sets.

D-KEFS is a neuropsychological battery which assesses a wide range of executive functions, such as cognitive flexibility, inhibition, planning, conceptualization, abstract thinking, and set shifting, and it consists of nine D-KEFS tests, including the Trail Making Test (TMT), Verbal Fluency Test, Design Fluency Test, Color-Word Interference Test (CWIT), Tower Test, Twenty Questions Test, Word Context Test, Sorting Test, and Proverb Test, with most of its tests widely used in the field of neuropsychology [5], individually or paired [6], using either relatively new or existing standardizations from other established clinical or experimental tests by other researchers [7]. For example, the CWIT was initially created by Stroop in 1935 [8], whereas TMT is a modification of the traditional test created by Brown and Partington in 1942 [7]. Each D-KEFS test gives primary as well as optional scores, which provide qualitative information about participants’ cognitive performance [9]. The inclusion of switching conditions constitutes a major characteristic of the D-KEFS among others [10]. In specific, these new switching conditions have been added to several of the D-KEFS tests, including the TMT, CWIT, Verbal Fluency TestOk and the Design Fluency Test. To sum, it is worth emphasizing that the D-KEFS is the first set of executive functioning tests, weighted in a large and representative national American sample, designed exclusively to assess executive functions [11].

### 1.2. CWIT_D-KEFS_

The CWIT_D-KEFS_ is one of the most widely used tools measuring processing speed, which is verbally mediated [12], as well as executive functions such as inhibition/switching skills, and cognitive flexibility, whereas it provides the ability to assess participants’ perseverance and tendency to act toward impulsive and unplanned responses in verbal modality. The basic requirement of the Stroop test is to assess participants’ ability to suppress a well-learned, automatic response (for example, word reading) in favor of a task which is not familiar (for example, naming the printed ink color of incongruously named colors) [13]. Therefore, the “Stroop interference effect” refers to the inhibition of a demanding task which prerequisites an automatic response, which is accompanied with a slower response speed as well as a lower accuracy of the incompatible task [14]. 

CWIT_D-KEFS_ constitutes a newer version of the Stroop Color and Word Test (SCWT; Stroop, 1935 [8]). Although research has previously focused on neuropsychological assessment in older adult populations with dementia [15], in recent years more focus has been given also to healthy populations as well as those adults with neurological and psychiatric conditions [15,16,17,18,19,20]. In this context, CWIT_D-KEFS_ has been used in healthy as well as clinical populations, for example, in young and older adults with traumatic brain injuries and frontal lesions [21,22,23,24], Parkinson’s disease [23], anorexia [25], frontal lobe or temporal lobe epilepsy [26], Autism spectrum disorder [27], cerebrovascular disease [28], substance use disorders [29], mild cognitive impairment due to Parkinson’s disease [30], and dementia due to various etiologies [31,32]. Hence, adapting widely used and highly complex neurocognitive tests contributes to the current focus of research and clinical practice.

### 1.3. TMT_D-KEFS_

The TMT is a modification of the traditional test created by Brown and Partington in 1942 [7], considered as one of the oldest, well-established and widely used neuropsychological tests. Therefore, it constitutes an inseparable part of the neuropsychological assessment administered to people with neurological and psychiatric conditions. A TMT assesses a set of high-level cognitive functions, such as switching/divided attention, inhibition, updating, cognitive flexibility, and set shifting [33], given that neuroimaging studies have found that frontal lobe dysfunction is highly related to executive function impairment [26,34,35]. Until now, the majority of studies, which will be mentioned below, use the traditional version of the TMT which includes only two conditions, number sequence and switching, instead of the TMT_D-KEFS_ which consists of five conditions including the previous from the traditional TMT version. Hence, it is quite important to extract norms for the TMT_D-KEFS_ to evaluate its clinical as well as research manifestations.

The TMT_D-KEFS_ is a traditional tool used to measure set-shifting (Letter–Number Switching) via including four baseline conditions (Visual Scanning, Number Sequencing, Letter Sequencing, and Motor Speed). Therefore, the TMT has been shown to be sensitive to the presence of brain injury and frontal lobe lesions [36,37], mainly on the Number–Letter Switching condition [38], autistic and Asperger’s disorder among adolescents and adult population [27], and psychiatric patients suffering from schizophrenia [39], whereas patients with frontal lobe epilepsy were disproportionately impaired compared with patients with temporal lobe epilepsy on the Number–Letter Switching condition (McDonald et al., 2005) [26]. Additionally, a recent study by Hacker et al. (2024) [40] showed that the TMT_D-KEFS_ could effectively detect patients with TBI among orthopedic populations and healthy participants. 

### 1.4. Demographics’ Effect: The Role of Age and Education

Delis et al. (2001) [41] assumed that age significantly affects participants’ performance across all D-KEFS tests. In specific, the longitudinal study by Adólfsdóttir et al. (2017) [18] demonstrated age-related changes in CWIT_D-KEFS_ performance, particularly in inhibition and switching among middle-aged and older adults, where the decline in performance of these abilities appeared to persist even after controlling for baseline test conditions, processing speed, test retest familiarity, gender, and years of education. Moreover, Zhao et al. (2020) [42] found that the switching condition of CWIT_D-KEFS_ was associated with age, which is also in agreement with previous studies that used the version of the CWST and Stroop test [43,44], respectively, whereas the homotopic connectivity of the ventromedial prefrontal cortex mediated the relationship between age and CWIT inhibition reaction time. Van der Elst et al. [12] reported that age-related decline was stronger for individuals with less education. On the other hand, Magnusdottir et al. (2021) [45] found that individuals with more education exhibited a stronger age-related decline. As regards the impact of gender, there is controversy across studies which found slightly better performance in women [45], while others found no gender effect [43,46]. 

According to studies [47,48], age and educational level as well as vocabulary have significant impacts on participants’ performances in the TMT, especially the subtests of letter sequencing and switching [47]. It seems that younger participants need significantly less time to complete the subtests in comparison to older participants [23,48,49,50], whereas older age and lower educational level are negatively related with satisfactory completion of the tests across all conditions [51]. To sum, regarding the TMT, age is assumed as the most important factor which affects participants’ performances [47], despite the controversy across studies which highlights education as the most predictive factor [47,52,53]. To sum, according to authors of the D-KEFS [41], age constitutes the predominant factor affecting performance across all D-KEFS subtests, and probably this is the reason why their initial normative data scores were stratified by age.

### 1.5. Study’s Aim

Until now, there have been almost no studies in Greece using D-KEFS in clinical and non-clinical populations; however, a few studies have administered D-KEFS to older Greek adults with vascular risk and subjective cognitive impairment, as well as clinical populations, in order to compare older adults with minor and major vascular problems with their healthy counterparts. All of them used the American raw scores to test their hypotheses [54,55,56,57]. To conclude, no previous studies using CWIT_D-KEFS_ and TMT_D-KEFS_ in a young adult population exist.

Finally, the already existing research concerning previous versions of the Stroop and TMT was conducted in healthy Greek adults by Zalonis et al. [44,48], who, however, did not use the D-KEFS version but mainly the traditional Stroop as well as TMT versions. Therefore, the TMT_D-KEFS_ and CWIT_D-KEFS_ have not been previously used with the Greek adult population in research or in clinical practice. No previous normative data study exists, because only American age-adjusted norms from the D-KEFS are available. Moreover, CWIT_D-KEFS_ and TMT_D-KEFS_ belong to those D-KEFS tests which are in common use, although their traditional versions (Stroop test and TMT) are used more commonly in neuropsychological assessment, which may reflect a lack of data in this field or a need to extend the relevant literature [58]. 

This present study aims to calculate norms in a typical Greek adult population based on age (20–29 years, 30–39 years, 40–49 years), to measure basic executive functions, and also to introduce this tool in clinical practice for the general population and patients with neurological and psychiatric conditions. Due to the relatively small sample size, this is an exploratory study and, therefore, more participants are needed to complete the data sample. As regards CWIT_D-KEFS_, results showed that age was the only predictor. As regards the TMT_D-KEFS_, age and educational level were predictors for most conditions, except for the visual scanning. Despite that, the initial version of the D-KEFS did not calculate separate norms stratified by educational level; we extracted norms matched for age and education. 

## 2. Materials and Methods

### 2.1. Participants

This study’s sample consisted of one hundred (101) healthy adults, aged from 20 to 49 years old (40% male and 60% female). The mean age was 32.21 years with a mean education of 14.98 years of schooling (Table 1). Participants were recruited using simple random sampling from the wider areas of North and South Greece, including towns, villages, and islands which belonged to these areas. In more detail, participants were university students from the University of Western Macedonia and Aristotle University of Thessaloniki, as well as other people from the aforementioned places of the country. Participants were enrolled in this study voluntarily, anonymously, and after giving their written consent. Before their inclusion in the study, participants read the information sheet which stipulated that the research team could use their data for research purposes. In order to extract the Greek norms, all participants were divided into three age classes, a typical separation in normative data studies (age range 20–29; 30–39, and 40–49 years), two gender classes (men and women), and three educational classes (secondary school final year and graduates: 10–12 schooling years; diploma degree, university students, bachelor’s degree: 13–16 years; and master’s degree, doctorate studies: 16< years).

Data collection was conducted by two psychologists, and each of them performed a different test in order to avoid any bias between raters. Test administration was conducted in a quiet place inside the university premises. 

The study’s inclusion criteria were the following: age from 20 to 49 years and Greek as their native language. The exclusion criteria were the following: presence of previous addictive disorder, psychosis and major depression, concurrent history of neurologic disease known to affect cognitive functioning, auditory functioning not sufficient to understand normal conversational speech, and visual acuity non-normal or non-corrected to anticipate visual stimuli.

### 2.2. Description of the D-KEFS Tests 

#### 2.2.1. CWIT

The Stroop test included three separate conditions: color naming speed, colors and words reading printed in black ink, and verbal responses’ inhibition through colors naming written in discordant ink. A fourth trial added to the CWIT_D-KEFS_ was named the inhibition/switching condition, which required a switch between color naming and word reading of color words printed in incongruent colors. Furthermore, the D-KEFS version appeared to use the Comalli variant [59], in which color naming precedes word reading. To sum, CWIT conditions were the following: color naming, word reading, inhibition, and inhibition/switching. 

In more detail, in the first condition the examinee was asked to name the colors they saw on the form (red, blue, green), hence, it measured naming speed in high-frequency repeating stimuli, e.g., color patches, as well as word-finding difficulty. The second condition required reading the color words, printed in black ink, and therefore it again measured the ability to read in high-frequency repeating stimuli. The third condition involves color naming, where the word and the color in which it was printed were incompatible with each other (e.g., the word “green” is printed in red ink), and thus the examinee needed to name the color that was printed and inhibit their automatic tendency that prompted them to read the word. Therefore, the examinee was required to name colors when inhibiting the more automatic task of reading the words. Lower performance in condition 3 was associated with deficits in verbal inhibition over the deficiency in naming speed. However, unlike other versions of this test, in the CWIT_D-KEFS_ there was also a fourth condition (the inhibition/switching condition), in which the examinee was asked to alternate at irregular intervals between reading color words and naming color words printed with discordant color [20]. Successful performance on this condition prerequisites naming speed, reading speed, cognitive flexibility, and verbal inhibition. This provided an extra requirement not only to inhibit the tendency to read but also to switch between different conditions through cognitive shifting [22]. In case the examinee performed well in conditions 1–3 but failed on condition 4, it implied deficits in cognitive flexibility, whereas problematic performance in both conditions 3 and 4 suggested impairments in verbal inhibition and cognitive flexibility. For each of the four conditions, the main score was based on the time required to complete the test. In addition, the number of uncorrected and self-corrected errors as well as the number of total errors in each condition were also measured. Finally, three primary contrast variables were also calculated: inhibition versus color naming, inhibition/switching versus combined naming plus reading, and inhibition/switching versus inhibition via subtracting the completion time scaled score for one or more component tasks from the completion time scaled score for one of the higher-level tasks in order. 

Findings showed an atypical pattern of performance, where most of the sample (57.1%) scored with faster completion time and/or fewer errors in the fourth condition than in the third, which may be attributed to two main factors. First, Lippa and Davis (2010) [60] argue that faster performance could be explained because color naming typically takes slightly longer than word reading, and the fourth condition involved only half as much color naming as the third. Second, the occurrence of fewer errors may be attributable to specific learning characteristics, such as increased verbal learning ability or increased semantic verbal fluency, which interacts with the order of test’s administration, i.e., the inhibition condition always precedes the inhibition/switching condition [60]. 

Finally, since Delis et al. (2001) [41] focused on the interpersonal connection and comfort between the examiner and the examinee during the neuropsychological assessment, Barnett et al. (2022) [61] studied the influence of them on the CWIT_D-KEFS_ performance. Findings showed that lower interaction between the examiner and the examinee was associated with increased completion time of inhibition condition; however, no differences were observed in inhibition/switching in relation to the interaction between them, which is perhaps explained by the fact that the third condition serves as a practical test for the suspension procedure in the inhibition/switching condition [60,61,62].

#### 2.2.2. TMT

The TMT_D-KEFS_ version provides a more detailed evaluation of neurocognitive performance by separating its conditions in control conditions (1, 2, 3, 5) which measure clinically relevant components of visuomotor sequencing skills as well as psychomotor speed, and on the other side, condition 4, which measures higher-order executive skills, for example, flexibility and cognitive set-shifting [63]. The main outcome measure is time-to-completion in seconds across all TMT_D-KEFS_ conditions. To conclude, the TMT_D-KEFS_ provides conceptual control conditions that provide the opportunity for detailed evaluation of neurocognitive performance through separating psychomotor speed and higher-order executive functions.

In detail, the first condition is named visual search and requires visual scanning. The second condition includes a numerical sequence and measures simple visual attention and visual scanning, visual-motor skills, and psychomotor speed. The third condition also consists of a visual-motor task and verbal learning task which measures grammatical sequences. The fourth condition measures the number–letter alternation assessing divided attention, cognitive flexibility, and set shifting by alternating two different sets of cognitive stimuli [64]. The fifth condition assesses motor speed [65]. Conditions 2 and 3 are often used to measure processing speed, while condition 4 is considered an index of cognitive function [65]. However, according to a previous study [16], the TMT_D-KEFS_ condition 4 was the only measure of executive functions which is clearly assumed as a functional ability index. To perform well in the switching tests, the examinee must have visual scanning and motor speed abilities to extract the score data, and the following test may describe problems in the above domains [63]. Lezak et al. (2012) [66] argue that the TMT is assumed as one of the most highly sensitive tools to detect neurocognitive deficits, and therefore, has an inseparable role in executive function evaluation. 

The first four conditions were presented on an A3 page, on which were scattered circles with numbers and letters as indicators. In condition 1 of visual scanning, the participant needed to delete the number 3 located in random order on the reference sheet among other numbers and letters. In the number sequence condition, the examinee needed to draw a single line sequentially connecting in ascending order numbers from 1 to 16, which were in random order on the reference sheet between letters. Correspondingly, in condition 3 of the letter sequence, the examinee needed to connect letters in order of the alphabet, from A to P, which were randomly presented among other numbers. In the number–letter alternation condition 4, the participant needed to draw a line, alternating the connections between numbers and letters sequentially. In more detail, starting from the number 1, the participant needed to draw a line to A, then from A to the number 2, from number 2 to the letter B, and so on until they reached the letter P. In the motor speed condition 5, the A3 reference sheet consisted of a single dotted line on which empty circles were scattered. The examinee needed to draw a line following the dotted line and passing through the circles located along the course (Table 2). Except for condition 4, which had a maximum time limit of 240 s, the remaining conditions had a limit of 150 s. In each case, the participant was asked to complete the tests as quickly as possible. If the participant made an error, the condition was interrupted, saying that an error had been made, and the examiner waited for the participant to find the error and continue the condition, without interfering with the stopwatch. Four out of the five basic test conditions can empirically show whether participants’ poor performances in the switching conditions is due to higher-level deficits in cognitive flexibility or due to impairments in the underlying skills needed to perform the switching tests, such as motor speed, visual scanning, and sequence of numbers or letters [47]. For example, poor performance in the alternation test may be due to deficits in visual search and motor control, whereas better performance in the letter sequence condition may contribute to better performance in the number–letter alternation condition [65].

### 2.3. Ethics

Consent was obtained from the Ethics Committee of the University of Western Macedonia to approve the processing of the participants’ data. Demographic information such as age, gender, and education, was collected, adhering to the law of the European Union since 28 May 2018, which allows the use of sensitive personal data for research purposes. Participants were told and consented that, upon a written request, their data could be removed from the online database. This study was aligned with the principles outlined in the Helsinki Declaration (World Medical Association, 1997).

### 2.4. Procedure

Initially, a pilot study was administered to a total of 10 people, mostly university students from the Psychology Departments, to evaluate CWIT and TMT_D-KEFS_ tests in order to avoid concomitant problems in data collection. Before their inclusion in the study, participants read the information sheet, which mentioned that the researchers could use their clinical data for research purposes. Additionally, they were told that they would be able to withdraw from the study whenever they wanted without facing any consequences or giving any explanation. Before the completion of the consent form, the participants were told that their records would be coded and anonymized for future research purposes. After signing consent to participate in the research, a short, structured interview followed to collect demographic information, including the participant’s gender, age, and education level. These data were accompanied by a code, which included the initial letters of the participant’s name in combination with the number of the series of administration (e.g., PM54) to preserve anonymity, but also to facilitate the identification process of the participants in the statistical database. D-KEFS test administration was conducted in a quiet environment at the university premises, mainly during morning hours, in order to perform better without external interference.

The neuropsychological assessment lasted about half an hour maximum and involved a face-to-face assessment. In specific, the instruction sheet of each condition was presented in front of the participants before each test’s administration. 

### 2.5. Statistical Analyses 

At first, an independent samples t test was conducted to detect possible gender effect on the two D-KEFS tests’ raw scores, as well as a Pearson correlation test for continuous variables to examine whether there was a correlation between age and education with the TMT_D-KEFS_ and CWIT_D-KEFS_ row scores. According to the literature review provided above, it seems that both tests are highly affected by age, especially when comparing younger with older-aged participants. However, since that these tests measure functions which are reduced during lifespan, it was of high significance to detect any age-effect even between very young individuals and those in early middle adulthood. Additionally, given that the initial normative sample from Delis et al. (2001) [41] includes age related norms, it seems that age impacts even younger age groups.

Despite the fact that the D-KEFS subtests’ raw scores were otherwise converted to scaled scores having a mean of 10 as well as a standard deviation of 3, in the current analyses we provided only raw scores to identify participants’ performances. Finally, inferential cut off scores were also calculated to select the score under which the possibility for an individual to belong to the normal population was below 10% and therefore would be assumed as low performance. The D-KEFS provided primary and optional variables in order to provide both global as well as more detailed evaluation about participants’ performances. In this current study, only primary measures were used, in agreement with similar studies in the literature. The primary scores attributable to each measure were as follows: CWIT_D-KEFS_ (the four conditions measuring time-to-completion, errors (corrected and uncorrected) across the four conditions and three contrast measures as also calculated by the Delis et al.’s [41] initial study, including inhibition versus color naming, inhibition/switching versus combined naming plus reading, and inhibition/switching versus inhibition), and TMT_D-KEFS_ (the five conditions measuring time-to-completion). 

An alpha value of 0.05 (two-tailed) was used. The statistical analyses were performed using the SPSS software v 27 (IBM Corp. Released 2020. IBM SPSS Statistics for Macintosh, Version 27.0. Armonk, NY, USA: IBM Corp).

## 3. Results

Demographic distribution is shown in Table 1. In line with the initial norms, the independent samples *t* test showed no gender classifications [CWIT1 *t* (98) = −0.210, *p* = 0.834; CWIT2 *t* (98) = −2.051, *p* = 0.043; CWIT3 *t* (98) = −0.088, *p* = 0.930; CWIT4 *t* (98) = 0.644, *p* = 0.521; TMT1 *t* (98) = 1.626, *p* = 0.107; TMT2 *t* (98) = 0.673, *p* = 0.503; TMT3 *t* (98) = 1.505, *p* = 0.136; TMT4 *t* (98) = −1.155, *p* = 0.251], in contrast to age, which was associated with both tests, and therefore their norms were stratified by age. Education was not correlated to the CWIT_D-KEFS,_ but instead the TMT_D-KEFS_, which was found to be significantly related to schooling years, specifically condition 4, which is the most complex and indicative of executive dysfunction, hence its norms were matched with age and education (Table 3 and Table 4). According to the results, CWIT errors, specifically total error score, corrected errors, and uncorrected errors did not differ by means of age and education years; however, in agreement with the initial study of Delis et al. [41], we calculated different norms across the three age groups of our study in order to provide a more concrete description of errors distribution in health experts.

Norms were established using percentiles scores (Table 5, Table 6, Table 7, Table 8, Table 9, Table 10, Table 11, Table 12 and Table 13). Specifically, we calculated the raw mean scores, as well as their standard deviation. Afterwards, we converted the raw scores into percentile scores. Inferential cut off scores were then calculated to extract those under the lowest 10%, which is the 10th percentile and is traditionally applied as low performance [67]. Scores above the 95% of the population were regarded as superior performance.

The CWIT norms for the four conditions were matched to age, color naming (*p* > 0.05), word reading (*p* < 0.05), inhibition (*p* < 0.01), and inhibition/switching (*p* < 0.01), but not stratified by gender and education because no differences were observed between men and women or between the three educational classes. On the contrary, the TMT_D-KEFS_ performance was strongly dependent on age and education, according to the Pearson test, and therefore, different age- and education-related norms have been extracted in each D-KEFS test’s condition: visual scanning (*p* > 0.05), number sequencing (*p* < 0.05), letter sequencing (*p* < 0.05), letter–number switching (*p* < 0.01) and motor speed (*p* < 0.01).

## 4. Discussion

To our knowledge, there are no norms outside the original American age-adjusted norms presented in the D-KEFS manual by Delis et al. (2001) [41] available for clinicians and researchers in Greece. Despite some previous versions of Stroop and the TMT validated in the adult Greek population, this is the first study which measures the effect of demographic variables on CWIT_D-KEFS_ as well as TMT_D-KEFS_ performance and provides normative data for the Greek sample. Moreover, there is a gap in the literature regarding neuropsychological tests’ normative data studies in the age range between early to middle adulthood, where no particular focus was given to the socio-demographic effect in examinees’ performances. This research gap is supposed to be covered by the current study.

Regarding the CWIT_D-KEFS_, it was created to improve the Stroop version by including an inhibition/switching trial, which was designed to be more difficult than the inhibition trial by means of completion time, as well as the number of errors. Overall, the results of the current study suggest that the CWIT_D-KEFS_ can be regarded as a measure of performance in processing speed/executive functioning, however, until now there have been no studies comparing the CWIT with the old Stroop test. The results of the current study showed that age was a predictive factor of CWIT_D-KEFS_ performance across its conditions, also in agreement with previous studies from Lippa and Davis (2010) [60], Zhao et al. (2020) [42], and Espenes et al. (2024) [68]. In fact, the three age classes of the sample differed by means of their performance in CWIT_D-KEFS_ mainly regarding the last two conditions. Based on the Pearson test, age classes differed statistically significantly in condition 3 (*r* = 0.489, *n* = 100, *p* < 0.001), 4 (*r* = 0.514, *n* = 100, *p* < 0.001), and 2 (*r* = 0.219, *n* = 100, *p* < 0.005). Additionally, regarding condition 2, it is worth mentioning that the average and upper percentile scores among the three age classes were identical with subtle differences, whereas those between 30 and 39 years old had a slightly better performance followed by those between 20 and 29 and those between 40 and 49 years old. However, this slightly lower performance of younger counterparts compared with adults who belong to the 30–39 age group does not necessarily mean lower executive functioning, because various factors, such as fatigue, low motivation, or reduced attention could affect their performance [69]. 

Kurniadi et al. (2021) [20] found moderate correlation between education with CWIT_D-KEFS_ performance in condition 3 (*r* = 0.212, *n* = 101, *p* < 0.005) and condition 4 (*r* = 0.319, *n* = 100, *p* < 0.001), whereas Karr et al. (2018; 2019 [70,71]) found that performance in the D-KEFS was proportional to participants’ level of education, which is in contrast to the results of the current study. Finally, regarding gender, no statistically significant correlation with performance was detected, which is confirmed by the study of Cutler et al. (2023) [24], in which the effect of gender did not play a significant role in the performance of the sample across all the tests’ conditions. Furthermore, in the longitudinal study by Adólfsdóttir et al. (2017) [18], a marginal correlation in performance was detected; as for the suspension condition, they predicted that the completion time would increase per year by 0.61% for men and by 0.62% for women, while for the suspension condition/shift, the rates would be 0.72% for men and 0.70% for women. 

The primary variable of the CWIT_D-KEFS_ was completion time, errors, and the three contrast measures mentioned previously. Therefore, norms at this stage of our study were calculated only for them, because they were assumed as primary scores. Norms for the optional scores, that include inhibition/switching versus color naming, as well as inhibition/switching versus word reading using their scaled scores, were not included in the current study. It is worth mentioning that in the first two conditions total errors were almost zero for all age groups without exception. Then, in condition 3, a limited number of errors were observed, most of which were self-corrected. This finding is probably related to the increase in completion time, relative to the previous baseline conditions, as correcting existing errors requires the participant to delay. Finally, in condition 4, the number of errors was twice as high as in condition 3, while the mean of uncorrected errors and self-corrections appeared almost equal, regardless of age. In fact, this finding agrees with the study of Lippa and Davis (2010) [60], where it was found that basically in people aged from 14 to 69 years, the average of errors was found greater in condition 4 than in 3. However, it contrasts with the study of Barnett et al. (2022) [61], because they argue that condition 3 is assumed to function as a practice test for condition 4 that requires inhibitory control and switching. In general, as the last two conditions are considered more complex, it was observed that the participants largely seemed to sacrifice more time in completing them, trying to avoid mistakes.

The inhibition/switching trial was designed to be the most difficult as regards completion time and the number of errors. Of particular interest is the study by Lippa and Davis (2010) [60], which investigates the complexity of the fourth condition compared to the third in adult population with an average level of 14.8 years of schooling and a diagnosis of either neurological or psychiatric pathology. Among people between 14 and 69 years old, the mean of errors in inhibition/switching condition was greater compared with the inhibition condition, whereas in those between 8 and 13 years old and 70 and 89 years old, the errors’ mean score was lower or equal with the ones in inhibition condition [60]. Moreover, longitudinal study of Adólfsdóttir et al. (2017) [18] demonstrated age-related changes in performance on inhibition, and combined inhibition and switching in middle-aged and older adults, where these populations had lower performance which appeared to persist, even after controlling for baseline measures of processing speed, gender, and years of schooling. Finally, norms for the three contrast variables—inhibition versus color naming, inhibition/switching versus combined naming plus reading, and inhibition/switching versus inhibition—were shown to be around 0 across all age groups, which is in line with the American norms.

A previous normative data study for the Stroop version called Trenerry’s Stroop Neuropsychological Screening Test (SNST) was conducted by Zalonis et al. (2009) [44] in a Greek adult population between 18 and 84 years and education range of 6–18 years of schooling. Contrary to our results, their findings suggested that both age and education significantly contributed to SNST performance. However, no direct comparisons can be made between our findings with the study of Zalonis et al. (2009) [44] because they included a broader sample in terms of age and education, which profoundly influenced examinees’ performances. Additionally, between those with 20–29 years old in the sample of this study, no one had less than 13 years of education, something that seems to be representative of the educational level of young adults in Greece according to the official laws of the government. Nevertheless, according to their findings, age appeared to be the most predictive factor of SNST performance, compared with education. Their study calculated four variables, including the color task, or the time needed to read the 112 items in the color–word task; the number of errors; the number of self-corrections; and the interference score, which were calculated by subtracting the number of errors from the total number of items completed in 120 s. On the contrary, in our study we followed a totally different scoring method via measuring the completion time across the four conditions. 

As regards the effect of demographics on the TMT_D-KEFS_, it is worth mentioning that most studies used the traditional version of the test, so although useful comparisons could be made, this controversy could be a limitation [51,72]. More specifically, previous studies which used the traditional version of the test in different populations showed that TMT performance appeared to be related to age and years of schooling [48,49,51,53,73], which is also confirmed by the findings of the current study. Except from the study of Fine et al. (2011) [47], the remaining studies that referred to the traditional version of the test showed that age and level of education were significantly related with the TMT execution time [48,49,53,73]. A possible explanation could be that typically, motor speed gradually declines with age [52], which is also in agreement with the study of Cavaco et al. (2013) [51], who found that the performance becomes better as the level of education increases. Finally, Fine et al. (2011) [47] showed that the effect of higher educational level is stronger mainly in the conditions of letter sequence and number–letter switching, which is also confirmed by the results of the current study. Although many studies found that overall age has a greater impact on the overall test performance than education [52], in our study we found that across the three age classes, those with 16+ years of schooling did better than their peers with a lower level of education, indicating the improvement in motor speed in those with a higher level of education, which is also confirmed by some previous studies [53]. 

Regarding gender, no differences were found by means of the TMT_D-KEFS_ performance in the current study. According to the literature review, previous studies observed differences between men and women. In specific, Cavaco et al. (2013) [51] found differences in the number sequencing condition, while a study by Cangoz et al. (2009) [72] reported a relationship between gender and TMT conditions. However, the study of Cangoz et al. (2009) [72] was conducted with people aged over 50, so no clear comparisons can be made. Heterogeneity of results by means of gender may be attributed to uncontrolled or unmeasured factors, such as sample’s characteristics, differences in men and women by means of educational level, and/or cultural differences [74]. 

Finally, according to the findings mentioned above, it seems that age and educational level may be predictive factors of TMT_D-KEFS_, because process speed declines with age [51,53] and increases with years of schooling [75]. These results are supported by theories about cognitive reserve which stress the protective role of education, among other factors, in cognitive decline, even in fluid intelligence aspects [76,77]. 

To compare the results of the current study with the first normative data study in a Greek adult population by Zalonis et al. (2009) [44], it was found that in their study they followed a totally different age clustering, because they divided their sample in 12 different groups (16–19, 20–29, 20–40, 25–45, 30–50, 35–55, 40–60, 45–65, 50–70, 55–75, 60–80, 65–85), and three were different from our educational levels according to the number of years of schooling (<9, 10–12, 13<). At this point, it must be noted that due to the absence of participants in the age group of 20–29 with educational level less than 10–12 years of schooling, the following category was not included in the normative tables. However, it is worth noting that the normative data scores in number sequence and switching conditions, which are common between the two TMT versions, was almost the same in the age class who had 10–12 years of schooling. Finally, given that the age classes between the two studies were very different, comparing them is vague and insufficient.

When comparing Greek norms of the CWIT with the American ones, stratified also by age, it was found that our mean scores belonged to the same range in terms of scaled scores which is equivalent to the mean American score of 10 or 1 SD above, which is acceptable according to what was previously mentioned. Hence, although Greek adults had an approximately slightly lower time-to-completion in some conditions, compared with Americans, this evidence can be attributed to higher educational levels across all age classes. Moving to the TMT_D-KEFS_, Greek norms were equivalent to Americans; however, in some conditions Greek adults scored 1 SD higher compared with the American sample, which can also be explained by the increased years of schooling. Finally, it can be assumed that the CWIT and TMT norms for the Greek adult population are equivalent to the original norms calculated by Delis et al. (2001) [41], hence they can be used by health professionals and researchers.

## 5. Limitations

The present research has some limitations that are worth mentioning. In particular, although an effort was made so that the sample corresponded to all levels of education, we did not manage to identify individuals exclusively with primary or secondary education for the age class of 20–29 years old. However, this seems to be representative of the Greek population. Moreover, another limitation was the limited sample size. Additionally, level of intelligence was not considered in the current study; however, given that all of them had more than 10 years of education, it can be assumed that intelligence was not a severe factor which could influence the current findings. Finally, both tests did not take into account the lexical characteristics of the sample, such as reading style or letter processing speed, differences which may have affected the processing of the Greek letter conditions in the visual-mental tracing.

## Figures and Tables

**Table 1:** Demographic distribution of the sample.

	Age Groups			Total
Years of Education	Age Group 20–29	Age Group 30–39	Age Group 40–49	
10–12	-	15	15	30
13–16	19	11	10	40
16+	11	11	10	31

**Table 2:** Ratio of letters between the English and Greek alphabets for conditions 1–4 of the TMT.

Numbers																
	1	2	3	4	5	6	7	8	9	10	11	12	13	14	15	16
Letters																
English	A	B	C	D	E	F	G	H	I	J	K	L	M	N	O	P
Greek	A	Β	Γ	Δ	Ε	Ζ	H	Θ	Ι	Κ	Λ	Μ	Ν	Ξ	O	Π

**Table 3:** Pearson correlation between time-to-completion on CWIT and demographical variables.

Variables	Age	Education
CWIT1 raw	0.031	−0.053
CWIT2 raw	0.219 *	−0.080
CWIT3 raw	0.489 **	−0.166
CWIT4 raw	0.514 **	−0.128

CWIT1 = color naming, CWIT2 = word reading, CWIT3 = inhibition, CWIT4 = inhibition/switching. * *p* < 0.05; ** *p* < 0.001.

**Table 4:** Pearson correlation between time-to-completion on TMT and demographical variables.

Variables	Age	Education
TMT1 raw	0.182	−0.036
TMT 2 raw	0.235 *	−0.142
TMT 3 raw	0.223 *	−0.142
TMT 4 raw	0.373 **	−0.240 *
TMT 5 raw	0.216 **	−0.267 **

TMT1 = visual scanning, ΤΜΤ2 = number sequencing, ΤΜΤ3 = letter sequencing, ΤΜΤ4 = number–letter switching, ΤΜΤ5 = motor speed. * *p* < 0.05; ** *p* < 0.001.

**Table 5:** Norms for the Trail Making Test (TMT) in conditions 1 to 5 in adults aged 20–29 years old.

	ΤΜΤ1	ΤΜΤ2	ΤΜΤ3	ΤΜΤ4	ΤΜΤ5	TMT1	TMT2	TMT3	TMT4	TMT5
%ile	13–16 Years of Education (Education-Class 2)					16< Years of Education (Education-Class 3)				
95	12.25	17.60	18.30	42.95	16.85	17.00	21.00	25.00	50.00	20.00
90	13.00	19.30	20.60	46.60	21.30	17.00	21.00	25.00	50.00	20.00
75	15.00	20.25	25.25	57.00	28.25	17.75	22.50	25.75	51.50	20.75
50	19.50	25.00	29.50	68.50	36.00	21.00	27.00	27.50	59.50	25.00
25	24.00	29.00	34.75	73.75	45.50	22.25	28.00	33.25	72.00	29.25
10	29.80	34.10	39.70	93.40	49.70	-	-	-	-	-
M.	20.47	25.31	30.34	67.94	36.00	20.33	25.67	28.83	61.17	25.00
S.D.	6.77	5.49	8.06	15.89	11.70	2.42	2.94	3.86	10.02	4.05

Notes: M = mean score, SD = standard deviation, TMT1 = visual scanning, ΤΜΤ2 = number sequencing, ΤΜΤ3 = letter sequencing, ΤΜΤ4 = number–letter switching, ΤΜΤ5 = motor speed.

**Table 6:** Norms for the Trail Making Test (TMT) in conditions 1 to 5 in adults aged 30–39 years old.

	TM1	TM2	TM3	TM4	TM5	TM1	TM2	TM3	TM4	TM5	TM1	TM2	TM3	TM4	TM5
%ile	10–12 Years of Education (Education-Class 1)					13–16 Years of Education (Education-Class 2)					16< (Education-Class 3)				
95	20.00	19.00	27.00	65.00	31.00	16.00	15.00	19.00	53.00	20.00	15.00	14.00	18.00	45.00	16.00
90	20.00	19.00	27.00	65.00	31.00	16.00	15.70	19.60	54.80	25.40	15.80	15.60	18.40	47.80	17.20
75	20.00	19.00	27.00	65.00	31.00	17.00	22.00	23.00	61.00	35.00	17.00	19.00	23.00	55.50	24.50
50	23.00	27.00	30.00	103.00	40.00	19.00	27.00	33.00	69.00	39.00	20.00	23.00	26.00	60.00	33.00
25	-	-	-	-	-	23.00	40.00	51.00	80.00	45.00	25.50	29.50	31.50	74.00	45.00
10	-	-	-	-	-	27.20	46.60	63.60	140.0	64.00	27.60	32.80	38.60	87.60	54.40
M.	23.00	27.00	29.33	91.67	43.33	20.00	29.53	37.67	79.20	40.80	21.38	24.15	27.00	64.77	34.54
S.D.	2.12	8.00	2.08	23.18	14.29	3.91	11.09	15.71	29.15	11.74	4.53	5.95	6.50	13.31	13.54

Notes: M = mean score, SD = standard deviation, TMT1 = visual scanning, ΤΜΤ2 = number sequencing, ΤΜΤ3 = letter sequencing, ΤΜΤ4 = number–letter switching, ΤΜΤ5 = motor speed.

**Table 7:** Norms for the Trail Making Test (TMT) in conditions 1 to 5 in adults aged 40–49 years old.

	TM1	TM2	TM3	TM4	TM5	TM1	TM2	TM3	TM4	TM5	TM1	TM2	TM3	TM4	TM5
%ile	10–12 Years of Education (Education-Class 1)					13–16 Years of Education (Education-Class 2)					16< (Education-Class 3)				
95	15.00	18.00	20.00	40.00	27.00	14.00	22.00	23.00	51.00	14.00	18.00	23.00	27.00	69.00	24.00
90	15.00	18.00	20.00	40.00	27.00	14.40	22.40	25.00	55.80	15.20	18.00	23.00	27.00	69.00	24.00
75	20.00	24.00	26.50	84.00	33.50	17.50	24.00	28.50	70.00	29.50	19.00	24.00	29.50	75.75	31.50
50	23.00	30.00	42.00	107.0	45.00	19.00	29.00	33.00	73.00	40.00	20.50	27.50	34.50	87.00	38.50
25	27.50	39.00	44.00	138.0	71.00	23.50	34.00	40.00	84.50	48.50	24.25	32.25	48.25	97.50	42.25
10	-	-	-	-	-	28.80	39.20	53.00	133.8	69.00	-	-	-	-	-
M.	23.78	31.78	36.22	106.6	50.89	20.38	29.69	35.23	80.23	40.08	21.63	27.88	38.25	86.63	37.75
S.D.	6.07	10.15	10.37	36.25	20.15	4.646	5.83	9.38	26.84	16.36	3.70	4.19	10.75	11.55	8.36

Notes: M = mean score, SD = standard deviation, TMT1 = visual scanning, ΤΜΤ2 = number sequencing, ΤΜΤ3 = letter sequencing, ΤΜΤ4 = number–letter switching, ΤΜΤ5 = motor speed.

**Table 8:** Norms for the Color-Word Interference (CWIT) in conditions 1 to 4 in adults aged 20–49 years old.

	Age Group 20–29				Age Group 30–39				Age Group 40–49			
%ile	CWIT1	CWIT2	CWIT3	CWIT4	CWIT1	CWIT2	CWIT3	CWIT4	CWIT1	CWIT2	CWIT3	CWIT4
95	21.00	16.05	33.05	39.05	16.10	16.10	31.65	44.00	20.55	16.55	37.75	47.75
90	21.20	17.00	35.10	43.00	20.00	17.10	37.30	48.10	21.20	17.10	42.30	52.00
75	24.00	18.00	37.00	47.00	24.00	19.00	44.75	52.75	24.75	19.00	49.75	56.75
50	25.00	20.00	41.00	51.50	25.00	20.00	49.50	60.00	25.00	21.00	56.00	64.00
25	29.75	22.00	50.00	57.00	29.00	21.25	57.75	68.25	30.00	23.25	62.00	70.00
10	30.90	23.90	59.80	63.60	30.90	22.90	60.90	80.00	31.90	26.00	65.00	80.00
M.	26.28	20.15	44.25	52	25.70	20.13	50.27	60.77	26.43	21.40	55.43	64.47
S.D.	3.78	2.66	9.44	8	4.24	2	9	10.45	3.67	3.26	8.44	9.89

Notes: M = mean score, SD = standard deviation, CWIT1 = color naming, CWIT2 = word reading, CWIT3 = inhibition, CWIT4 = inhibition/switching.

**Table 9:** Norms for the Color-Word Interference (CWIT) for the CWIT1 in errors (total score, corrected, and uncorrected) in adults aged 20–49 years old.

	Age Group 20–29			Age Group 30–39			Age Group 40–49		
%ile	Uncorr	Corrected	Total	Uncorr	Corrected	Total	Uncorr	Corrected	Total
95	00.00	00.00	00.00	00.00	00.00	00.00	00.00	00.00	00.00
90	00.00	00.00	00.00	00.00	00.90	00.90	00.00	00.90	00.90
75	00.00	00.00	00.00	00.00	00.00	00.00	00.00	00.00	00.00
50	00.00	00.00	00.00	00.00	00.00	00.00	00.00	00.00	00.00
25	00.00	00.00	00.00	00.00	00.00	00.00	00.00	00.00	00.00
10	00.00	00.00	00.00	00.00	00.90	00.90	00.00	00.90	00.90
M.	00.00	00.05	00.05	00.00	00.10	00.10	00.00	00.10	00.10
S.D.	00.00	00.22	00.22	00.00	00.30	00.30	00.00	00.30	00.30

Notes: M = mean score, SD = standard deviation, CWIT1 = color naming, Uncorr = uncorrected errors, Corrected = corrected errors, Total = total errors.

**Table 10:** Norms for the Color-Word Interference (CWIT) for the CWIT2 in errors (total score, corrected, and uncorrected) in adults aged 20–49 years old.

	Age Group 20–29			Age Group 30–39			Age Group 40–49		
%ile	Uncorr	Corrected	Total	Uncorr	Corrected	Total	Uncorr	Corrected	Total
95	00.00	00.45	00.00	00.00	00.00	00.00	00.00	00.00	00.00
90	00.00	00.00	00.00	00.00	00.00	00.00	00.00	00.00	00.00
75	00.00	00.00	00.00	00.00	00.00	00.00	00.00	00.00	00.00
50	00.00	00.00	00.00	00.00	00.00	00.00	00.00	00.00	00.00
25	00.00	00.00	00.00	00.00	00.00	00.00	00.00	00.00	00.00
10	00.00	00.00	00.00	00.00	00.00	00.00	00.00	00.00	00.00
M.	00.03	00.00	00.03	00.00	00.00	00.00	00.00	00.03	00.03
S.D.	00.15	00.00	00.15	00.00	00.00	00.00	00.00	00.18	00.18

Notes: M = mean score, SD = standard deviation, CWIT2 = word reading, Uncorr = uncorrected errors, Corrected = corrected errors, Total = total errors.

**Table 11:** Norms for the Color-Word Interference (CWIT) for the CWIT3 in errors (total score, corrected, and uncorrected) in adults aged 20–49 years old.

	Age Group 20–29			Age Group 30–39			Age Group 40–49		
%ile	Uncorr	Corrected	Total	Uncorr	Corrected	Total	Uncorr	Corrected	Total
95	0.00	0.00	0.00	0.00	0.00	0.00	0.00	0.00	0.00
90	0.90	0.00	0.00	0.00	0.00	0.00	0.00	0.00	0.00
75	0.00	0.00	0.00	0.00	1.00	1.00	0.00	1.00	1.00
50	0.00	1.00	1.00	0.00	1.00	1.00	0.00	1.25	2.25
25	0.00	2.90	3.00	0.00	2.00	0.00	1.00	3.00	3.00
10	1.00	3.00	3.95	0.45	2.00	0.00	2.45	3.00	3.45
M.	0.10	0.73	0.83	0.03	0.73	0.77	0.23	0.93	1.17
S.D.	0.30	1.08	1.23	0.18	0.69	0.67	0.67	1.04	1.28

Notes: M = mean score, SD = standard deviation, CWIT3 = inhibition, Uncorr = uncorrected errors, Corrected = corrected errors, Total = total errors.

**Table 12:** Norms for the Color-Word Interference (CWIT) for the CWIT4 in errors (total score, corrected, and uncorrected) in adults aged 20–49 years old.

	Age Group 20–29			Age Group 30–39			Age Group 40–49		
%ile	Uncorr	Corrected	Total	Uncorr	Corrected	Total	Uncorr	Corrected	Total
95	0.00	0.00	0.00	0.00	0.00	0.00	0.00	0.00	0.00
90	0.00	0.00	0.10	0.00	0.00	0.00	0.00	0.00	0.00
75	0.00	1.00	1.00	0.00	0.00	0.00	0.00	0.00	1.00
50	0.00	1.00	2.00	0.00	1.00	1.00	1.00	1.00	2.00
25	1.00	2.00	3.00	1.00	2.00	3.00	2.00	2.00	4.00
10	2.90	3.00	4.00	1.00	3.00	3.90	3.00	3.00	5.90
M.	0.78	1.40	2.15	0.40	1.13	1.53	1.27	1.27	2.53
S.D.	1.09	1.00	1.47	0.56	1.13	1.38	1.28	1.11	1.97

Notes: M = mean score, SD = standard deviation, CWIT4 = inhibition/switching, Uncorr = uncorrected errors, Corrected = corrected errors, Total = total errors.

**Table 13:** Norms for the composite scaled score equivalents of scaled scores by all age groups.

%ile	Inhibition vs. Color Naming	Inhibition/ Switching vs. Combined Naming + Reading	Inhibition/Switching vs. Inhibition
95	3.00	2.00	2.00
90	3.00	1.00	2.00
75	2.00	1.00	0.75
50	0.00	−1.00	−1.00
25	−2.00	−2.00	−2.00
10	−3.00	−4.00	−3.00
M.	−0.21	−0.98	−0.56
S.D.	2.32	2.09	1.88

Notes: M = mean score, SD = standard deviation.

## Data Availability

Data is unavailable due to privacy and ethical restrictions.
